# Development of a New Salt of Piperine with Toluene Sulfonic Acid and Its Anti-Inflammation Effect In Vivo

**DOI:** 10.3390/molecules29235631

**Published:** 2024-11-28

**Authors:** Ilma Nugrahani, Ari Sartinah, Hidehiro Uekusa, Yuto Abekura, Slamet Ibrahim, Kusnandar Anggadiredja

**Affiliations:** 1 School of Pharmacy, Bandung Institute of Technology, Bandung 40132, Indonesia; kusnandar_a@itb.ac.id; 2 Center of Halal Studies, Bandung Institute of Technology, Bandung 40132, Indonesia; 3 Faculty of Pharmacy, Halu Oleo University, Kendari 93231, Indonesia; 4 School of Science, Tokyo Institute of Technology, Tokyo 152-8551, Japan; uekusa@chem.titech.ac.jp (H.U.); abekura98921masato183@gmail.com (Y.A.); 5 Faculty of Pharmacy, Jenderal Achmad Yani University, Cimahi 40531, Indonesia; slametibrahim@yahoo.com

**Keywords:** organic salt, piperine, para-toluene sulfonic acid (TSA), solubility, anti-inflammation

## Abstract

Piperine (PPN) is a natural compound with an anti-inflammation effect and low solubility. Hence, some molecular modifications have improved its solid-state character, including cocrystal formation. However, the salt structure has yet to be widely studied. In this research, PPN was reacted with toluene sulfonic acid (TSA), which was expected to increase its solubility and anti-inflammatory effects. This experiment used solid-state reactions and analysis, including thermal analysis, infrared spectroscopy, and powder X-ray diffractometry, to characterize the new multicomponent solid phase. Next, single crystal R-ray diffractometry was used to determine the final three-dimensional conformation structure. After that, the salt, which can be reproduced by wet grinding, was tested for improved stability and anti-inflammatory effects. As a result, the PPN–TSA multicomponent solid was confirmed as a solid salt with monoclinic packing, exhibiting better solubility and anti-inflammation effects. Thus, this new organic salt has the potential for further phytochemical compound development.

## 1. Introduction

Inflammation is the most common symptom in unhealthy conditions, and it is associated with pain in many stages. To treat this condition, anti-inflammatory agents, including steroids and non-steroidal anti-inflammatory drugs (NSAIDs) [1], are widely used [2]. Unfortunately, many unexpected responses and side effects on gastrointestinal, renal, cardiovascular, and hepatic function are related to those anti-inflammatory drugs [3,4]. Hence, more attention is being given to natural medicines, including piperine, to replace drugs with minimum undesired effects [5].

Structurally, piperine (PPN) is an alkaloid from the *Piperacaeae* plant [6] that showed some kind of activity. This compound has been developed as an anti-osteoarthritis and anti-rheumatoid arthritis agent, as well as a common anti-inflammatory agent [7,8,9]. Unfortunately, this substance has low solubility, ~40 mg/L in water [10], which is predicted to diminish the bioavailability and pharmacological effect [11]. Therefore, efforts are being made to improve the physicochemical properties, including composing a multicomponent system [12]. Several developed structures that used succinic acid [13] and saccharin [14] have been reported to increase the solubility of PPN. On average, the derived molecules were neutral multicomponents/cocrystals. We recently also reported a new neutral multicomponent system of piperine with a weak anti-inflammatory agent, hydroxybenzoic acid, showing a synergetic effect and significantly increasing the natural compound’s solubility and anti-inflammation activity [15].

**Figure 1:**
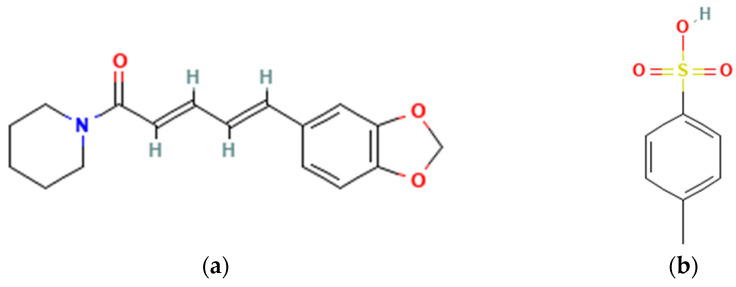
Molecular structure of parent compounds: (**a**) piperine [17] and (**b**) para-toluene sulfonic acid [18].

Meanwhile, the salt form has not been developed much. Therefore, this experiment aimed to arrange a multicomponent salt system of PPN with a stronger acid counterion, para-toluene sulfonic acid (TSA), and investigate its solubility and anti-inflammatory activity. The salt system was expected to increase the solubility of PPN and then the anti-inflammatory activity. As known, salting is an ionic reaction between an acid and a base [16]. Based on its structure in Figure 1a, PPN is a base (pKa: 12.2) [17]. This, it was expected to interact ionically with an acid moiety, TSA, with the structure shown in Figure 1b, which has a pKa = −1.34 [18] and has been used in the pharmaceutical industry [19]. Recent research reported that TSA is a counterion to febuxostat (FEB), an anti-hyperuricemia drug, and a multicomponent compound, FEB-TSA, was produced that exhibited a fivefold increase in FEB solubility [20].

Furthermore, a single crystal from slow evaporation was isolated for structural determination using a single crystal X-ray diffractometer (SCXRD). Meanwhile, a green method, solvent-dropped grinding, was used to prepare the salt with a higher mass volume, as confirmed by a series of solid-state analysis instruments. After that, a solubility test was done, and following ethical clearance, an anti-inflammation test was performed on Wistar rats in vivo.

## 2. Results

### 2.1. Molar Ratio Screening

Screening of the selected molar ratio was carried out by looking at the narrowest or sharpest melting temperature range of the three molar ratios of the PPN and TSA reaction mixture, namely 1:2, 1:1, and 2:1. The study results showed that the 1:1 molar ratio had the sharpest melting temperature range, which indicated that this molar ratio was the most suitable stoichiometric ratio for the formation of multicomponent salts. The molar ratio screening results are shown in Supplementary Table S1.

### 2.2. PPN–TSA Single Crystal

**Figure 2:**
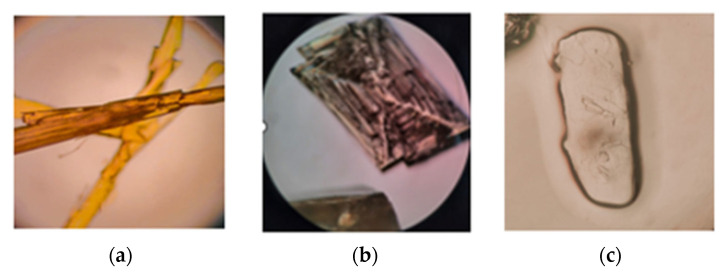
The crystal form of PPN–TSA (**a**) compared to PPN (**b**) and TSA (**c**).

The crystal yielded by mixing, dissolving, and evaporating the solution of PPN–TSA at a 1:1 molar ratio in 95% ethanol is shown in Figure 2a and compared to the single crystals of PPN and TSA in Figure 2b and Figure 2c, respectively.

### 2.3. Solid-State Characterization

**Figure 3:**
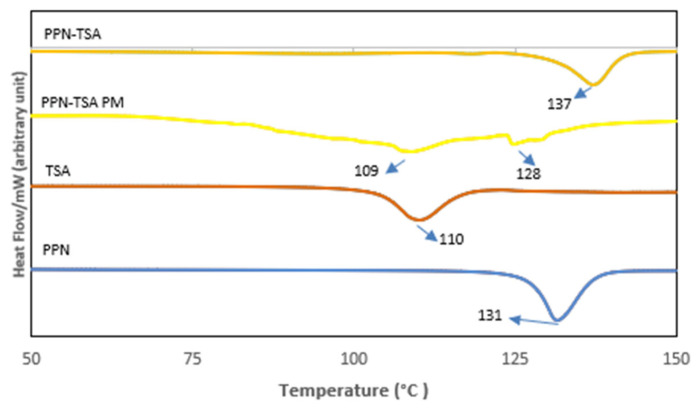
DSC thermogram of PPN–TSA salt compared to its constituent components PPN (piperine), TSA (p-toluene sulfonic acid), and the physical mixture PPN–TSA PM.

First, electrothermal data showed a melting point of 137 °C that matched with the DSC thermogram in Figure 3.

**Figure 4:**
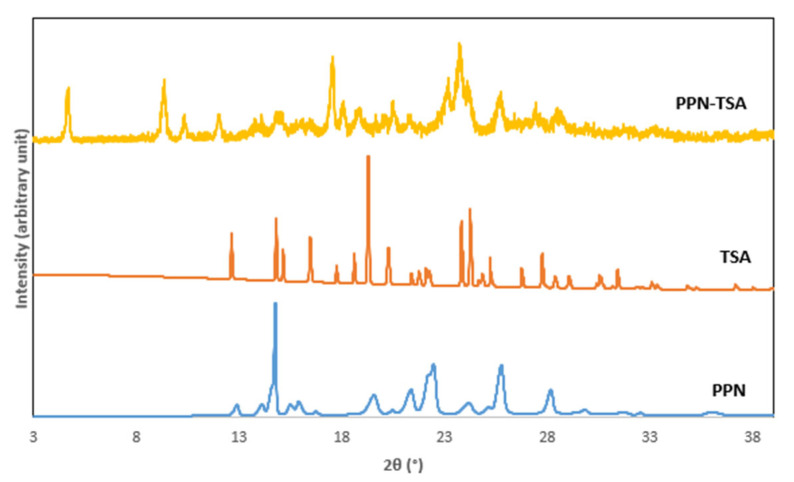
PXRD diffractogram of PPN–TSA salt and its constituent compounds (PPN/piperine and TSA/p-toluene sulfonic acid).

Next, powder X-ray diffractometry (PXRD) confirmed the formation of a new phase. Figure 4 presents the diffractogram of the PPN–TSA multicomponent system compared to PPN and TSA.

### 2.4. Structural Study

#### 2.4.1. FTIR

**Figure 5:**
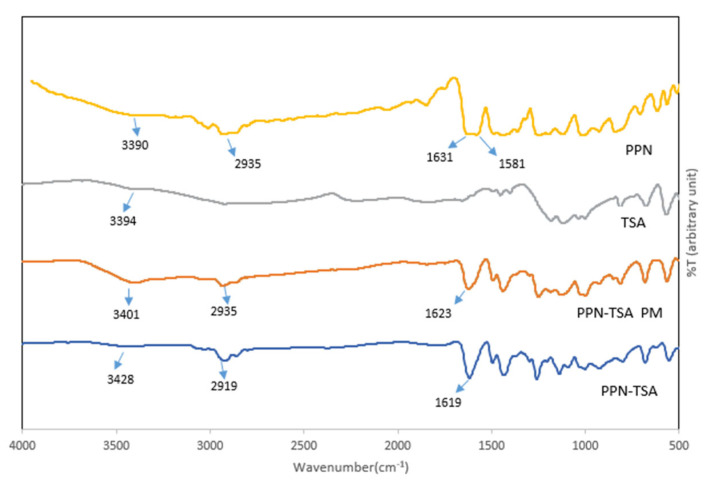
FTIR spectra of PPN–TSA (piperine–toluene sulfonate) compared to piperine, TSA (p-toluene sulfonic acid), and PM (physical mixture).

First, the FTIR spectra in Figure 5 elucidated the reaction in two dimensions.

#### 2.4.2. SCXRD

**Figure 6:**
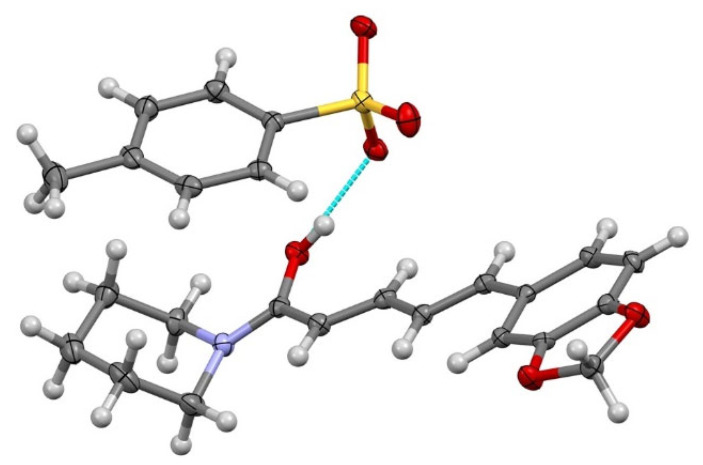
Thermal ellipsoid drawing of PPN–TSA (piperine–toluene sulfonate) with a probability of 50%. The hydrogen bond is shown as a light blue line.

**Figure 7:**
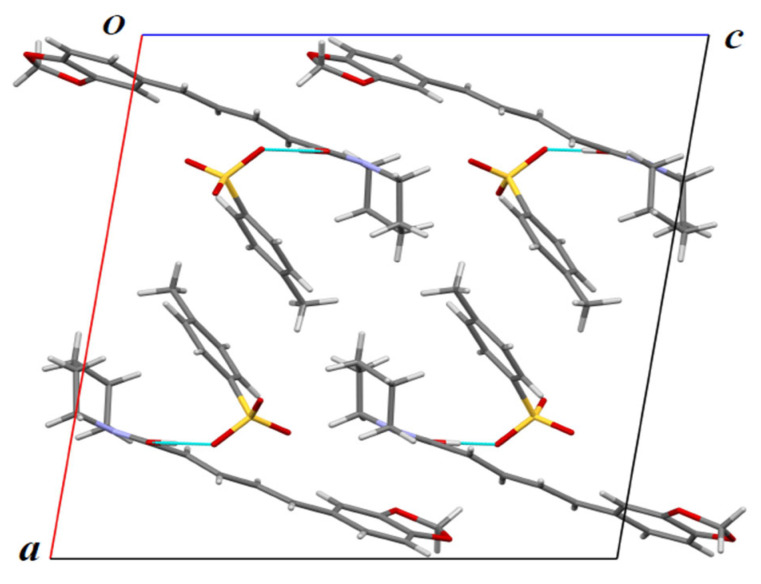
Packing drawing of PPN–TSA (piperine–toluene sulfonate) viewed along the b-axis. A hydrogen bond is shown as a light blue line.

SCXRD analyzed the three-dimensional single crystal and produced a structure of PPN–TSA in Figure 6 with the packing drawing shown in Figure 7.

**Figure 8:**
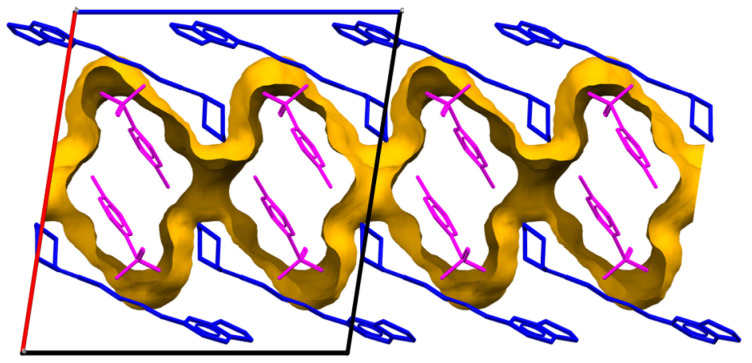
Channel drawing was viewed along the b-axis. Piperine is depicted in blue, and toluene sulfonate is denoted in magenta. The TSA channel appears as golden walls.

**Figure 9:**
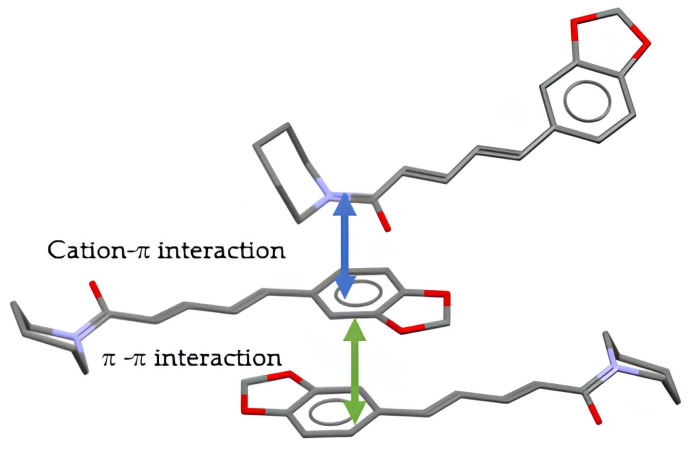
Stacking of piperine with cation–π (blue) and π–π interactions (green). H atoms are omitted for clarity.

Next, Figure 8 depicts the b-axis view of the channel-like TSA, and Figure 9 shows the stack of benzene rings.

Supplementary Table S2 includes complete information on crystallographic data and the related CIF file and CheckCIF.

### 2.5. Solubility Data

**Figure 10:**
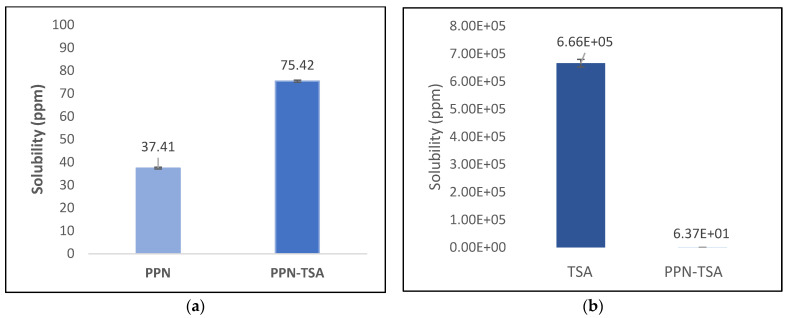
Solubility of (**a**) piperine from PPN–TSA (piperine–toluene sulfonate) salt and alone (PPN); (**b**) TSA (p-toluene sulfonic acid) in PPN–TSA salt and TSA in the water media; *n* = 3.

In this study, we reported that PPN’s solubility in the salt increased, as shown in Figure 10a,b.

### 2.6. Anti-Inflammatory Activities

**Figure 11:**
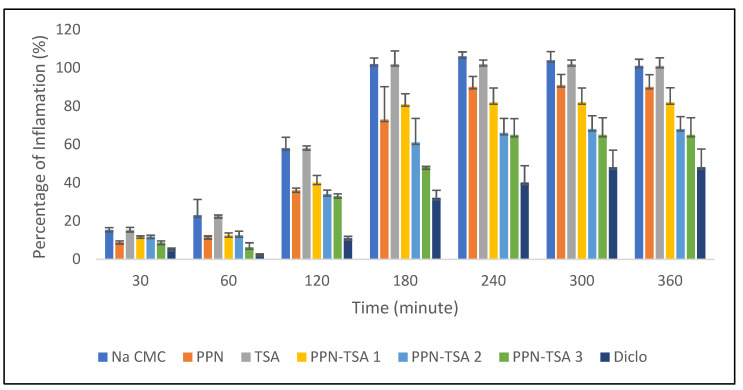
The percentage of inflammation in the groups of rats with carrageenan-induced inflammation administered Na CMC 1%, diclo (diclofenac sodium doses 4.5 mg/Kg BW), PPN (piperine doses 50 Kg/BW), TSA (p-toluene sulfonic acid doses 33 Kg/BW), and PPN–TSA (piperine–toluene sulfonate salt) in three dose variations (PPN–TSA 1 dose at 21 mg/Kg BW; PPN–TSA 2 doses at 42 Kg/BW; PPN–TSA 3 doses at 83 Kg/BW). All data were checked with one-way ANOVA, which showed significant response differences between groups, followed by the Turkey test.

The following is the inflammatory response presented as the percentage of inflammation in Figure 11, which was composed based on data in Supplementary Table S3.

## 3. Discussion

Previously, we reported a neutral interaction between PPN and hydroxybenzoic acid (HBA), causing the solubility to increase and enhancing the anti-inflammation effect. HBA also has an anti-inflammation impact. In this case, the combination of PPN and HBA showed synergism. In this experiment, PPN was reacted with a strong acid with no activity, TSA, to form a salt, hoping that the solubility might be improved. Then, its anti-inflammatory activity was investigated. First, the screening was done using a diagram phase as a simple method for determining the stoichiometric balance, indicating that the area between two eutectic points corresponded to the 3:7 and 7:3 melting points. The data are provided in Supplementary S1. Hence, the 2:1, 1:1, and 1:2 molar ratios were tested for reaction with the assistance of a solvent. However, only the PPN–TSA (1:1) product indicated distinct solid-state characteristics based on its visual appearance (Figure 2), melting point and thermal character (Figure 3), diffractogram (Figure 4), and infrared spectra (Figure 5), without any evidence of starting material remaining.

First, Figure 2 showed the different visuals of the PPN-TSA system with the starting components. The PPN-TSA is needle-like, while the PPN and TSA are rectangular. Next, the thermogram in Figure 3 presents an endothermic PPN–TSA thermal profile, indicating that the salt melted at 137 °C. Meanwhile, the physical mixture has irregular endothermic curves at 109 °C and 128 °C with a broader peak.

Figure 4 shows the diffractogram of the PPN–TSA crystal, with distinct diffraction peaks at 2θ = 4.69°, 9.35°, 10.35°, 12.02°, and 17.55° that are significantly different from the constituent compounds. Hereafter, considering the new crystal features, the thermogram and the diffractogram physically confirmed the formation of a new solid-state phase. The physical characterization related to the latest chemical entity was then thoroughly evaluated with FTIR and SCXRD.

PPN–TSA’s FTIR spectra, depicted in Figure 5, had different bands than its starting material and the mixture before the reaction. Structurally, PPN has benzene rings, C–O, and a C–N moiety, forming a long conjugation system with C=C bonds [21]. Next, the typical peaks at 3008 cm^−1^, 1581 cm^−1^, 1488 cm^−1^, and 1446 cm^−1^ were caused by the -CH stretching vibration of the aromatic ring. Also, the C=C and C=O stretching vibrations are observed at 2935 and 1631 cm^−1^, respectively. Meanwhile, the characteristic peaks of TSA appear at 3394 cm^−1^ (OH) and 1600 cm^−1^ (aromatic C=C) [21]. Spectral changes occurred when the PPN–TSA salt was formed, as indicated by the C=O shift at 1631 cm^−1^ and 1619 cm^−1^. In addition, the –OH stretch shifted from 3394 cm^−1^ to 3428 cm^−1^. This spectral shift indicates the interaction between the C=O of PPN and the OH of TSA.

Next, SCXRD was used to assess the salt structure, revealing the distances between oxygen atoms of the carboxylic acid moiety of TSA and the cationic group of PPN. Based on the data in Figure 6, the solid-state compound was composed of a 1:1 ratio of PPN and TSA, with a proton of the SO_3_H group of the organic acid transferred to the amide oxygen of PPN to form the salt, creating an OH…O^—^ charge-assisted strong hydrogen bond (O…O 2.5149(13) Å). C–OH length in the generated piperinium cation was increased to 1.308(2) Å from the usual C=O length (1.22–1.24 Å) (Figure 6). Next, Figure 7 depicts the three-dimensional structure of the packing system of the new salt.

The TSA anions in the crystals were aligned to form a channel-like structure along the b-axis (Figure 8). After that, Figure 9 shows that the amide N^+^ moiety of PPN was stacked on the benzene ring of PPN with cation–π interactions (N…benzene plane: 3.392 Å); meanwhile, the benzene rings were stacked with π–π interactions (benzene plane distance is ±3.42 Å). Completing the crystal information in Supplementary S2, it can be concluded that this multicomponent is a salt, which differs from the recently reported neutral interaction (cocrystal) between piperine and hydroxybenzoate [15]. This is due to the use of a stronger acid in this experiment than the previous one. In some cases, salt formation can increase solubility in water [22,23], which was then evaluated using a solubility test.

After the solid analysis and structure determination, the salt’s solubility was compared to that of the parent compound, PPN. The solvent evaporation method required time. Hereafter, solubility and anti-inflammation tests used the wet grinding technique or solvent-dropped grinding using ethanol 95% for scaling up to yield grams of the product. This technique was conducted to make the process efficient and produce a homogenous salt particle [24,25]. In line with the expectation, the reaction quickly occurred using this green method, yielding the salt powder in grams. The product obtained matched the single crystal from the solvent evaporation with similar product characteristics, including the melting point, FTIR profile, and diffractogram.

Figure 10a shows that the solubility of PPN in the salt form increased twofold compared with its parent component. In general, charged ions can interact strongly with polar molecules, especially water in solution; therefore, salts are often very soluble in water. Likewise, charge-assisted solid hydrogen bonding interactions usually hold salts together, as in the PPN–TSA salt structure, which frequently creates a large portion of one or more ions with exposed polar functionality [26]. These hydrogen bonding interactions usually make most ions possess open polar functionality, thus increasing the solubility in water. Furthermore, as shown in Figure 8, the TSA anion is in a one-dimensional channel in the crystal. It easily desorbs water, which destabilizes the crystal and causes it to collapse. This feature of the crystal structure makes PPN–TSA easy to dissolve. Increased solubility will determine the amount of absorption in biological systems, thus also increasing its pharmacological effect [27]. On the other hand, TSA’s solubility in the salt form decreased significantly compared to its single form and became equal to PPN, as shown in Figure 10b.

The anti-inflammatory activity of a substance is measured by its ability to reduce the level of edema produced by the inducer in animal tests. Carrageenan is an inflammatory inducer that can stimulate the release of inflammatory and pro-inflammatory mediators [28]. Figure 11 reveals that PPN–TSA had better anti-inflammation activity than PPN alone. Based on Figure 11 and data in Supplementary S4, administration of the PPN–TSA salt inhibited the inflammation 30 min after carrageenan administration. This increased anti-inflammatory effect is thought to occur due to increased solubility. However, the graphic in Figure 11 shows no significant difference in the anti-inflammatory activity between PPN–TSA doses 2 and 3 after 120 min. Increasing solubility will influence the next steps in diffusion and absorption. This will increase bioavailability and improve the anti-inflammatory effect [15,29,30]. Another factor to consider was the permeability properties. Structurally, the blood–brain barrier as a drug receptor is composed of lipophilic lipids [31]. PPN is lipophilic [32], making it easier to penetrate the blood–brain barrier than hydrophilic substances, so it activates better.

However, compared to the PPN cocrystal form with hydroxybenzoic acid (Pip–HBA) [15], PPN–TSA salt has slightly lower anti-inflammation activity. This phenomenon can be predicted by the solubility of the PPN–TSA salt, which was a little bit less soluble than the Pip–HBA cocrystal form. The increase in PPN–HBA solubility was threefold, while the solubility of the PPN–TSA salt was increased twofold compared with PPN. In addition, HBA had anti-inflammatory activity, but TSA did not. In the regulation, substances with activity should be considered more, but the inert one is simpler. However, some studies, i.e., toxicity and pharmacokinetic profile, can be done to complete the information of this newly developed salt. Nevertheless, this new salt expands the multicomponent structure catalog in the Cambridge Structural Database (submitted under number 2356771), offering solubility and pharmacological benefits. In addition to oral administration, this compound may also be developed in a topical dosage form with a specific solubility and penetrated enhancer.

## 4. Materials and Methods

### 4.1. Materials

Piperine, with a grade of >95%, was bought from Synaptent, LLC (Chicago, IL, USA). Next, p-toluene sulfonic acid >99%, sodium carboxymethyl cellulose, and carrageenan came from the Tokyo Chemical Industry (Tokyo, Japan). The 95% grade of ethanol was purchased from Sakura Medical (Bandung, Indonesia); meanwhile, potassium bromide/KBr was purchased from Merck (Jakarta, Indonesia). Distilled water was prepared by the Bandung Institute of Technology (Bandung, Indonesia). The aluminum plates and lids for DSC measurement were purchased from Rigaku (Tokyo, Japan), and the capillary tube for electrothermal analysis was prepared by CV Prima Medica (Bandung, Indonesia). Male Wistar rats, *Rattus norvegicus*, were bred and collected from the School of Life Sciences and Technology, Bandung Institute of Technology (Bandung, Indonesia).

### 4.2. Methods

#### 4.2.1. Molar Ratio Determination

The fixed molar ratio of the targeted compound was investigated by measuring the melting temperature range using Electrothermal AZ 9003 (Staffordshire, UK). The temperature data were then plotted against the composition to make a phase diagram. The 1:1, 1:2, and 2:1 compositions were selected and analyzed, and the fit molar ratio should correspond to the sharpest melting temperature.

#### 4.2.2. Single Crystal Preparation

The fixed molar ratio of PPN–TSA was found to be a (1:1) molar ratio. A single crystal for structural determination was isolated using a slow evaporation method by dissolving the fit molar ratio PPN–TSA mixture entirely in 95% ethanol and filtering. Next, the solution was put into an Erlenmeyer to be evaporated at room temperature until the appropriate crystals grew. The PPN–TSA crystal was characterized using thermal analysis (electrothermal and DSC), PXRD, and FTIR to confirm the new solid-state formation. After that, an appropriate crystal was analyzed using SCXRD.

#### 4.2.3. Upscaling of the Multicomponent by Wet Grinding

##### Salt Scaling Up

The scaling-up technique for increasing the PPN–TSA multicomponent system was grinding with 95% ethanol dropping in a small volume. The (1:1) molar ratio mixture was prepared in ~5 g and ground homogeneously in a mortar assisted by a stamper. Next, the powder was dropped in 5 mL of 95% ethanol and ground until forming a wet mass. It was ground for ~10 min and let dry at room temperature. The treatment was repeated three times, and it was left to dry. Samples were stored as the wet grinding product and characterized using the same solid-state analysis instruments, and the product should be similar to the single crystal [24,25].

#### 4.2.4. Solid-State Characterization and Structure Determination

First, a binocular microscope Olympus CX21 (Tokyo, Japan) was used to observe the PPN, TSA, and PPN–TSA salt features. Next, the melting point was measured with an Electrothermal AZ 9003 (Staffordshire, UK), and DSC Rigaku Thermoplus EVO2 DSC8231 (Tokyo, Japan) was employed to collect the thermogram. After that, PXRD Rigaku Miniflex (Tokyo, Japan) was used to confirm the new solid-state arrangement. After that, FTIR Jasco 4200 Type-A (Oklahoma City, OK, USA) was used to read the new interactions as a sign of a new chemical structure formation. Finally, the SCXRD Rigaku R-AXIS RAPID II diffractometer with VariMax 007 from Rigaku OD (Tokyo, Japan) thoroughly solved the salt’s three-dimensional structure.

- *Microscope Observation*

PPN, TSA, and PPN–TSA crystals were observed under a binocular microscope at 10× magnification. The images of crystals were taken using a OPPO A3s mobile camera (Dongguan, China).

- *Electrothermal Analysis*

A small sample was filled into a capillary tube for electrothermal analysis and placed in the holder. The measurement was set at 30 ◦C and 10 °C/min heating rates. The melting point was observed through the observation hole [33].

- *Differential Scanning Calorimetry (DSC)*

Approximately 2–5 mg sample was ground homogenously, put into an aluminum pan, closed by pressing the lid, and placed in the sample holder. On the other side, an empty aluminum pan was used as a reference. The measurement was done from room temperature to 250 °C, with a rate of 10 °C/min. Then, the thermal profile data were processed to produce a thermogram using Microsoft Excel 365 software (Washington, DC, USA) [34].

- *Powder X-ray Diffractometry (PXRD)*

About 50 mg of sample powder was placed in the holder. The measurement was conducted at 2θ with an interval of 3–50° and a scanning rate of 5°/min, using Cu-Kα radiation with a graphite monochromator. The diffractogram was drawn using Microsoft Excel 365 and Origin software 2024 [35].

- *Fourier Transform Infrared (FTIR) Analysis*

The sample was mixed homogenously with KBr (as the background) with a ratio of 1:100 *w*/*w*. It was then put into a sample plate and compacted using a hydraulic press to produce a clear pellet, which was then put into the holder and scanned along a wavelength of 4000–400 cm^−1^ [36].

- *Single Crystal X-ray Diffractometry (SCXRD)*

A single crystal’s acceptable and appropriate size (~0.4–0.5 mm) was carefully selected under a binocular microscope, and the crystal was put into the sample holder. That crystal sample was held on a goniometer head and put in a diffractometer for the measurement. Analysis was done in ω-scan mode with Cu-Kα radiation with a λ = 1.54184 Å and X-ray optics as the rotating anode. The study was done under –180 °C. The diffraction pattern was integrated and scaled using RAPID AUTO (Rigaku, 2009) software (Rigaku, Tokyo, Japan). Meanwhile, the direct structural elucidation was processed using SHELXT and refined with SHELXL [37,38]. In this three-dimensional structure determination, a different Fourier map was taken for the hydrogen atoms, which were placed by the riding model for geometrical calculations during the refinement. On the other side, the non-hydrogen atoms were refined anisotropically. Finally, the structure was composed using the software Mercury 4.3.1 [39].

#### 4.2.5. UV Spectrophotometry Derivative Method Development

The derivation was done mathematically to separate the spectra of PPN from TSA, which dissociated in the solution. First, PPN and TSA absorption were scanned, and then the mixture’s solution spectra were compared. Due to overlapping spectra, the derivation was done to look for the appropriate spectrum for PPN concentration determination. The calibration curve was composed of a series of the mixture’s concentration of the solution standard, which then was derived mathematically using the embedded software in the instrument until a specific wavenumber was found. The validation parameters were evaluated, especially the linearity, which should have r > 0.990 [40]. This method determined PPN concentration in the solubility test and assay.

#### 4.2.6. Solubility Testing

Each sample was dissolved in 50 mL water in an Erlenmeyer, put in a shaker, stirred until saturated, and filtered. The concentration of that saturated solution, named the solubility value, was measured using the validated derivative UV spectrophotometry [41] in triplicate.

#### 4.2.7. Anti-Inflammatory Activity

- Animals

The male Wistar rats (*Rattus norvegicus*) were weighed. Rats at 150–200 g were treated following ethical clearance. The selection of male animals is based on the consideration that male rats have hormonal stability compared to female rats. Those rats were maintained under standard laboratory conditions with a light period of 12 h/day and a temperature of 27° C ± 2° C. The availability of food and water was maintained.

- Carrageenan-induced paw inflammation

The rats were divided into seven groups, each group consisted of 5 animals. First, the negative control, group 1, was only given Na-CMC, and the positive control (group 2) was given diclofenac sodium as the reference drug at a dose of 4.5 mg/kg BW. Next, group 3 was administered PPN at 50 mg/kg BW, and group 4 was administered TSA at 33 mg/kg BW. After that, the testing groups were orally administered a series of PPN–TSA doses: group 5 = 21 mg/kg BW (PPN–TSA 1), group 6 = PPN–TSA 42 mg/kg BW (PPN–TSA 2), and group 7 = PPN–TSA 83 mg/kg BW (PPN–TSA 3).

Briefly, 0.1 mL carrageenan suspension (1% solution in 0.9% saline solution) was injected subcutaneously into the sub-plantar on the left hind paw 30 min after administering the testing solution samples orally. After that, the change in paw volume caused by edema after 30, 60, 120, 180, 240, 300, and 360 min was measured using a plethysmometer. The degree of inflammation was determined by calculating the percentage difference in paw edema before and after drug administration [42].

### 4.3. Statistics

All data from analysis instruments were independently obtained from triplicate trial results and presented as the means of the data with the standard deviation value. Microsoft Excel (Microsoft Corp., Wahington, DC, USA) assisted in composing graphics. Furthermore, one-way ANOVA and the Tukey test were utilized to determine the significant differences in the anti-inflammatory data among groups.

## 5. Conclusions

Preparation, characterization, and determination of the structure of PPN–TSA salt have been successfully carried out. Based on a solubility test, the PPN–TSA salt was two times more soluble in water than PPN. Consequently, it had better anti-inflammatory activity than PPN. Improving the solubility of PPN using multicomponent crystallization is a difficult task, but a new technique in which PPN arranges a salt with strong acids accomplishes the task. This technique allows salt formation in many APIs that are thought to be unable to form salts.

## Data Availability

Data are included in the article as Supplementary Material or are referenced in the article. The crystal structure has been deposited in CCDC (Cambridge Crystallography Data Center) under number 2356771, which contains the supplementary crystallographic data for this paper. The data can be freely obtained from the Cambridge Crystallographic Data Centre via www.ccdc.cam.ac.uk/structures.

## Supplementary Materials

The following supporting information can be downloaded at: https://www.mdpi.com/article/10.3390/molecules29235631/s1, Table S1: Molar ratio screening; Table S2: Crystal data and structure refinement for PPN–TSA; Table S3: Percentage of inflammation of sodium carboxymethyl cellulose (Na CMC), diclofenac sodium (diclo), piperine (PPN), p-toluene sulfonic acid (TSA), and PPN–TSA (three dosage variations) on carrageenan-induced inflammation.
