# Supplementary material for: Development of a New Salt of Piperine with Toluene Sulfonic Acid and Its Anti-Inflammation Effect In Vivo
Source: Molecules. 2024 Nov 28;29(23):5631. doi: 10.3390/molecules29235631 (PMC11643923; doi:10.3390/molecules29235631)

checkCIF/PLATON report

Structure factors have been supplied for datablock(s) shelx_a

THIS REPORT IS FOR GUIDANCE ONLY. IF USED AS PART OF A REVIEW PROCEDURE FOR PUBLICATION, IT SHOULD NOT REPLACE THE EXPERTISE OF AN EXPERIENCED CRYSTALLOGRAPHIC REFEREE.

No syntax errors found. CIF dictionary Interpreting this report

Datablock: shelx_a

```
Bond precision:   C-C = 0.0019 A          Wavelength=1.54186

Cell:             a=18.4650(3)     b=6.8851(1)        c=17.3870(3)
                  alpha=90         beta=98.785(1)     gamma=90
Temperature:      93 K

                Calculated                   Reported
Volume          2184.54(6)                   2184.54(6)
Space group     P 21/c                       P 21/c
Hall group      -P 2ybc                      -P 2ybc
Moiety formula  C17 H20 N O3, C7 H7 O3 S     C17 H20 N O3, C7 H7 O3 S
Sum formula     C24 H27 N O6 S               C24 H27 N O6 S
Mr              457.53                       457.52
Dx,g cm-3       1.391                        1.391
Z               4                            4
Mu (mm-1)       1.674                        1.674
F000            968.0                        968.0
F000'           972.26
h,k,lmax        22,8,20                      22,7,20
Nref            4004                         3946
Tmin,Tmax       0.787,0.844                  0.752,0.849
Tmin'           0.752

Correction method= # Reported T Limits: Tmin=0.752 Tmax=0.849
AbsCorr = MULTI-SCAN

Data completeness= 0.986           Theta(max)= 68.245

R(reflections)= 0.0346( 3663)                          wR2(reflections)=
                                                       0.0923( 3946)
S = 1.105                    Npar= 291
```

The following ALERTS were generated. Each ALERT has the format
test-name_ALERT_alert-type_alert-level.
Click on the hyperlinks for more details of the test.

Alert level C

```
PLAT410_ALERT_2_C Short Intra H...H Contact  H11      ..H17A     .       1.97 Ang.
                                                   x,y,z  =      1_555 Check
PLAT911_ALERT_3_C Missing FCF Refl Between Thmin & STh/L=    0.600          6 Report
PLAT918_ALERT_3_C Reflection(s) with I(obs) much Smaller I(calc) .          1 Check
```

Alert level G

```
PLAT007_ALERT_5_G Number of Unrefined Donor-H Atoms .............           1 Report
PLAT066_ALERT_1_G Predicted and Reported Tmin&Tmax Range Identical          ? Check
PLAT398_ALERT_2_G Deviating  C-O-C   Angle From 120 for O18      .      105.8 Degree
PLAT398_ALERT_2_G Deviating  C-O-C   Angle From 120 for O19      .      106.1 Degree
PLAT480_ALERT_4_G Long H...A H-Bond Reported H1B      ..O23      .       2.61 Ang.
PLAT883_ALERT_1_G No Info/Value for _atom_sites_solution_primary .     Please Do !
PLAT910_ALERT_3_G Missing # of FCF Reflection(s) Below Theta(Min).          1 Note
PLAT912_ALERT_4_G Missing # of FCF Reflections Above STh/L=  0.600          1 Note
PLAT978_ALERT_2_G Number C-C Bonds with Positive Residual Density.         17 Info
```

0 **ALERT level A** = Most likely a serious problem - resolve or explain
0 **ALERT level B** = A potentially serious problem, consider carefully
3 **ALERT level C** = Check. Ensure it is not caused by an omission or oversight
9 **ALERT level G** = General information/check it is not something unexpected

2 ALERT type 1 CIF construction/syntax error, inconsistent or missing data
4 ALERT type 2 Indicator that the structure model may be wrong or deficient
3 ALERT type 3 Indicator that the structure quality may be low
2 ALERT type 4 Improvement, methodology, query or suggestion
1 ALERT type 5 Informative message, check

It is advisable to attempt to resolve as many as possible of the alerts in all categories. Often the minor alerts point to easily fixed oversights, errors and omissions in your CIF or refinement strategy, so attention to these fine details can be worthwhile. In order to resolve some of the more serious problems it may be necessary to carry out additional measurements or structure refinements. However, the purpose of your study may justify the reported deviations and the more serious of these should normally be commented upon in the discussion or experimental section of a paper or in the "special_details" fields of the CIF. checkCIF was carefully designed to identify outliers and unusual parameters, but every test has its limitations and alerts that are not important in a particular case may appear. Conversely, the absence of alerts does not guarantee there are no aspects of the results needing attention. It is up to the individual to critically assess their own results and, if necessary, seek expert advice.

Publication of your CIF in IUCr journals

A basic structural check has been run on your CIF. These basic checks will be run on all CIFs submitted for publication in IUCr journals (*Acta Crystallographica*, *Journal of Applied Crystallography*, *Journal of Synchrotron Radiation*); however, if you intend to submit to *Acta Crystallographica Section C* or *E* or *IUCrData*, you should make sure that full publication checks are run on the final version of your CIF prior to submission.

Publication of your CIF in other journals

Please refer to the *Notes for Authors* of the relevant journal for any special instructions relating to CIF submission.

PLATON version of 28/11/2022; check.def file version of 28/11/2022

Datablock shelx_a - ellipsoid plot

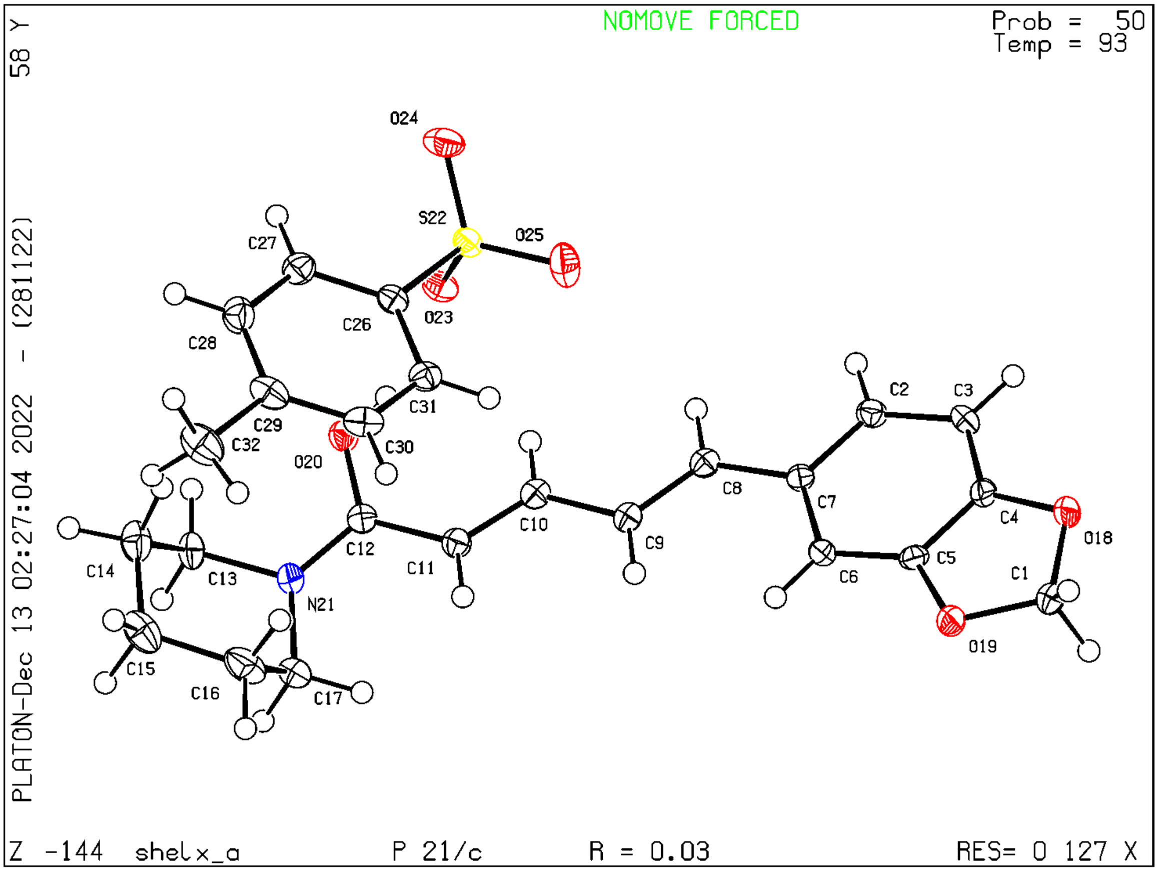

Supplement: Supplementary file 1 [file molecules-29-05631-s001.zip › checkcif.pdf]
