# Supplementary material for: Development of a New Salt of Piperine with Toluene Sulfonic Acid and Its Anti-Inflammation Effect In Vivo
Source: Molecules. 2024 Nov 28;29(23):5631. doi: 10.3390/molecules29235631 (PMC11643923; doi:10.3390/molecules29235631)
Supplement: Supplementary file 1 [file molecules-29-05631-s001.zip › Supplementary_S1_S2_S3.pdf]

Table S1. Molar Ratio Screening

No.	Molar Ratio	Melting point (°C)
1.	1:2	109-131
2.	1:1	135-138
3.	2:1	110-130

Table S2. Crystal data and structure refinement for PPN-TSA

Parameters	PPN-TSA	
Empirical formula	$C_{24} H_{27} N O_6 S$	
Formula weight	457.52	
Temperature	93(2) K	
Wavelength	1.54186 Å	
Crystal system	Monoclinic	
Space group	$P2_1/c$	
Unit cell dimensions	a = 18.4650(3) Å	
	b = 6.8851(1) Å	β= 98.785(1)°
	c = 17.3870(3) Å	
Volume	2184.54(6) Å^3	
Z	4	
Density (calculated)	1.391 g/cm^3	
Absorption coefficient	1.674 mm^{-1}	
F(000)	968	
Crystal size	0.170 x 0.119 x 0.101 mm^3	
Theta range for data collection	4.847 to 68.245°.	
Index ranges	-22<=h<=22, -7<=k<=7, -20<=l<=20	
Reflections collected	38485	
Independent reflections	3946 [R(int) = 0.0388]	
Completeness to theta = 67.686°	98.7 %	
Absorption correction	Semi-empirical from equivalents	
Max. and min. transmission	0.849 and 0.752	
Refinement method	Full-matrix least-squares on F^2	
Data / restraints / parameters	3946 / 0 / 291	
Goodness-of-fit on F^2	1.105	
Final R indices [I>2sigma(I)]	R_1 = 0.0346, wR_2 = 0.0909	
R indices (all data)	R_1 = 0.0363, wR_2 = 0.0923	
Largest diff. peak and hole	0.489 and -0.255 e.Å^{-3}	
CCDC Deposit Number	2356771	

Table S3. Percentage of inflammation of sodium carboxymethyl cellulose (Na CMC), diclofenac sodium (diclo), piperine (PPN), p-toluene sulfonic acid (TSA), and PPN-TSA (three dosage variations) on carrageenan-induced inflammation

Samples	Percentage of Inflammation (mean±SD %)						
	T1/2	T1	T2	T3	T4	T5	T6
Na CMC	15.363±1.2	23,667±8.3	58.33±5.7	102.667±3.1	106.33±2.3	104.667±4.5	102.00±3.5
PPN	9.02±0.6	11.667±0.6	36.667±1.2	73.333±17.2	90.333±5.5	90.333±5.5	91.333±6.4
TSA	15.306±1.4	22.667±0.6	58.667±1.2	102.33±6.8	102.33±2.1	102.33±2.1	101.667±4.2
PPN-TSA 1	12.00±0.2	12.667±1.2	40.667±3.8	81.3333±5.5	82.333±7.5	82.333±7.5	82.667±7.6
PPN -TSA 2	11.91±0.7	12.667±2.1	34.667±2.1	61.667±12.6	66.667±7.6	68.667±7.0	68.333±6.5
PPN -TSA 3	8.71±0.9	6.333±2.3	33.333±1.2	48.00±6.0	65.333±8.5	65.667±9.0	65.667±9.0
Diclo	5.47±0.4	2.667±0.2	11.00±1.0	32.667±4.0	40.00±8.9	38.333±9.0	38.333±9.6
